# Post-intubation tracheal lacerations: Risk-stratification and treatment protocol according to morphological classification

**DOI:** 10.3389/fsurg.2022.1049126

**Published:** 2022-11-23

**Authors:** Giuseppe Cardillo, Sara Ricciardi, Anna Rita Forcione, Luigi Carbone, Francesco Carleo, Marco Di Martino, Massimo O. Jaus, Salvatore Perdichizzi, Marco Scarci, Alberto Ricci, Raffaele Dello Iacono, Gabriele Lucantoni, Giovanni Galluccio

**Affiliations:** ^1^ Unit of Thoracic Surgery, Azienda Ospedaliera San Camillo-Forlanini, Carlo Forlanini Hospital, Rome, Italy; ^2^ Unicamillus–Saint Camillus University of Health Sciences, Rome, Italy; ^3^ PhD Program, Alma Mater Studiorum, University of Bologna, Bologna, Italy; ^4^ Management Department, University of Bologna, Bologna, Italy; ^5^ Department of Cardiothoracic Surgery, Imperial College Healthcare NHS Trust, London, United Kingdom; ^6^ Unit of Pulmonology, Sapienza University of Rome, San Andrea Hospital, Rome, Italy; ^7^ Unit of Pulmonology and Thoracic Endoscopy, Azienda Ospedaliera San Camillo-Forlanini,; ^8^ Carlo Forlanini Hospital, Rome, Italy

**Keywords:** tracheal laceration, post-intubation, conservative treatment, morphological classification, surgery, fibrin glue

## Abstract

**Background:**

Post-intubation tracheal laceration (PITL) is a rare condition (0.005% of intubations). The treatment of choice has traditionally been surgical repair. Following our first report in 2010 of treatment protocol tailored to a risk-stratified morphological classification there is now clear evidence that conservative therapy represents the gold standard in the majority of patients. In this paper we aim to validate our risk-stratified treatment protocol through the largest ever reported series of patients.

**Methods:**

This retrospective analysis is based on a prospectively collected series (2003–2020) of 62 patients with PITL, staged and treated according to our revised morphological classification.

**Results:**

Fifty-five patients with Level I (#8), II (#36) and IIIA (#11) PITL were successfully treated conservatively. Six patients with Level IIIB injury and 1 patient with Level IV underwent a surgical repair of the trachea. No mortality was reported. Bronchoscopy confirmed complete healing in all patients by day 30. Statistical analysis showed age only to be a risk factor for PITL severity.

**Conclusions:**

Our previously proposed risk-stratified morphological classification has been validated as the major tool for defining the type of treatment in PITL.

## Introduction

Despite the large number of tracheal intubations performed every day, tracheal lacerations are extremely rare (0.005% of intubations), and generally involve the pars membranacea of the cervico-thoracic trachea in the midline (1).

The prevalence of PITL in elective intubations ranges from 1 in 20,000 to 75,000 patients (2–10) while in emergency procedures it is estimated to occur in up to 15% of cases (11). The report of PITL is higher (0.5%–1%) for double-lumen intubation (5, 10, 14–16). PITL may represent a life-threatening condition that requires prompt diagnosis, management, and treatment (11–14).

Risk factors for tracheal ruptures include multiple attempts at forced intubation, inexperience of the healthcare provider (e.g., anaesthesiologist) attempting the intubation, endotracheal tube introducers that protrude beyond the tip of the tube and inappropriate use of a stylet (17, 18). Patient-related factors that increase the risk for tracheal injury and rupture include congenital tracheal abnormalities, weakness of the pars membranacea of the trachea, chronic obstructive pulmonary disease and other inflammatory lesions of the tracheobronchial tree (19), advanced age and female gender (20–22). Symptoms of tracheal injury include soft tissue or mediastinal emphysema, pneumothorax, dyspnoea, and haemoptysis.

In the evaluation of treatment strategies for PITL several parameters should be considered such as presence of pneumothorax, stabilization of vital signs, respiratory status (either spontaneous or mechanical), bronchoscopy findings, and CT scan imaging.

Surgery has traditionally represented the cornerstone of PITL treatment, with most clinicians recommending surgical repair in the first instance of PITL in the hope that early surgical management prevents the potential lethal complications of PITL, mainly mediastinitis and tracheal stenosis. Conservative treatment was usually reserved for the minority of patients with small, haemodynamically stable tracheal lesions with a length of tracheal damage <3 cm (1, 2, 5, 7, 13), with the decision making depending mostly on the local expertise and no consensus or standardized treatment.

In 2010 we presented an original morphological classification for patients-risk-stratification in which the key element to guide surgeons in the choice of treatment of PITL (conservative vs. surgery) was the depth instead of the length of the tracheal injury (11).

The present study reports our overall series of 62 patients with PITL and represents an internal validation of our previously reported morphological classification in order to standardise the approach and develop clinical management protocols for tracheal lacerations (11). An external validation of our classification is also reported from the literature.

## Materials and methods

This retrospective analysis is based on a prospectively collected series treated at Azienda Ospedaliera San Camillo Forlanini in Rome. All patients had provided informed consent prior to any procedures. Approval by the institution ethics committee was not required as dictated by local laws.

A total of 32 new patients of PITL were identified between December 2008 and January 2020. These cases were combined for internal validation with 30 cases that occurred from January 2003 to November 2008 and were reported in our previously published paper (11).

**Figure 1:**
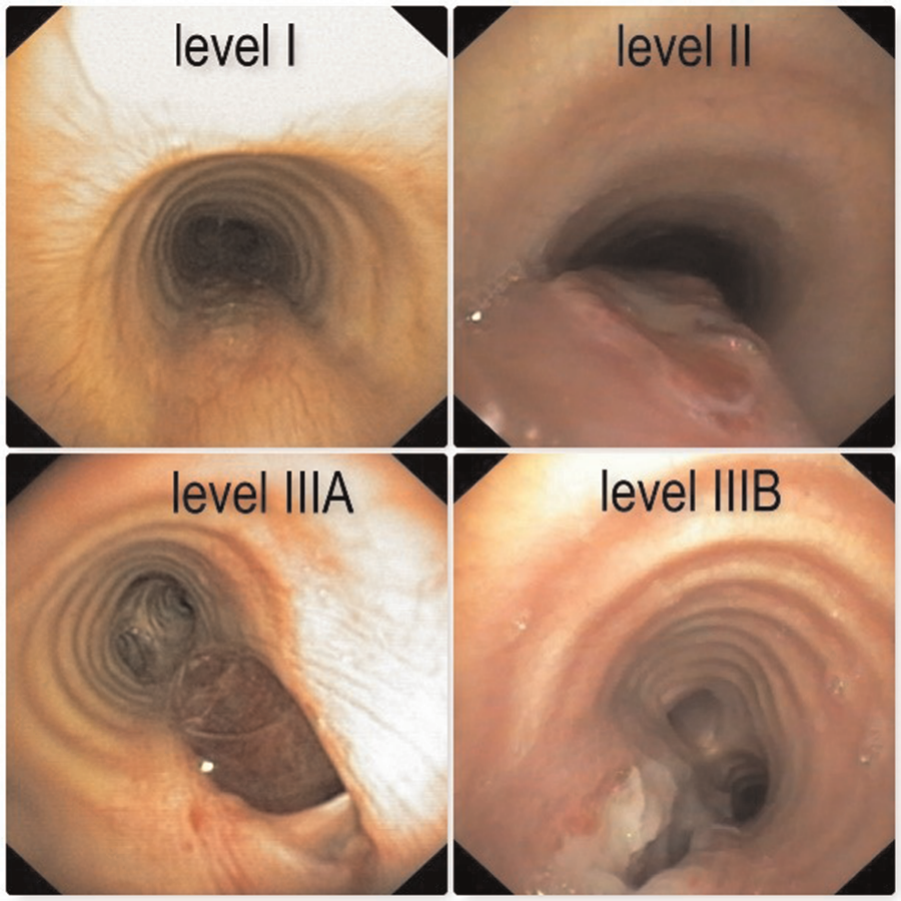
Photograph depictions of PITL level I, level II, level IIIA and level IIIB.

**Figure 2:**
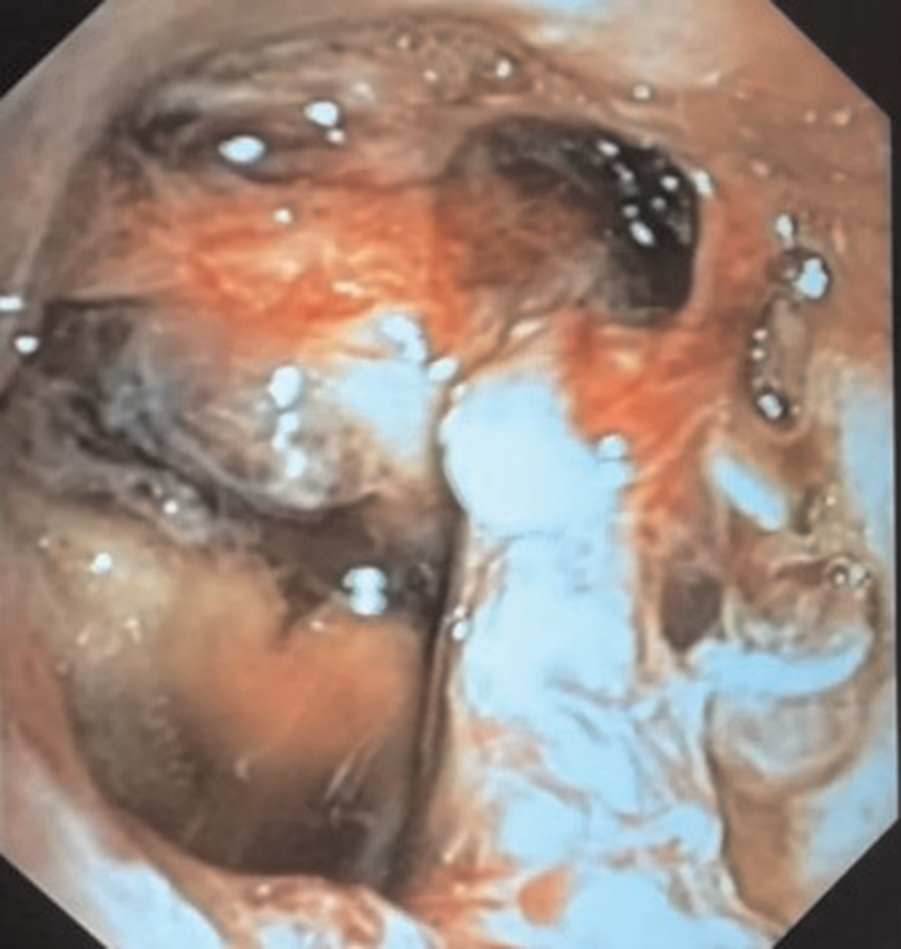
Photograph depictions of PITL level IV.

All patients with suspicious PITL underwent early bronchoscopy (within 48 h) to identify lesion site and extent, including the length and location of the tracheal tear, with careful assessment of the upper and lower lesion limit, lesion morphology, and depth of transmural involvement: depending on the depth of the tracheal wall involvement, PITL lesions were staged using the Cardillo's revised morphologic classification listed in Table 1 and shown in Figures 1, 2. The revised classification, compared to the previous, added a new stage, PITL Level IV**,** which represents an Extensive Loss of substance/fracture of tracheal rings.

**Table 1:** Cardillo's revised morphologic classification of the tracheal injury.

Classification #	Morphologic description
Level I	Mucosal/Submucosal tracheal involvement without subcutaneous -mediastinal emphysema (partial thickness PITL)
Level II	Full-thickness tracheal lesion with subcutaneous or mediastinal emphysema without oesophageal injury or mediastinitis
Level IIIA	Full-thickness laceration of the tracheal wall with oesophageal or mediastinal soft-tissue hernia without oesophageal injury or mediastinitis
Level IIIB	Full-thickness laceration of the tracheal wall with oesophageal injury or mediastinitis
Level IV	Extensive Loss of substance/fracture of tracheal rings

Modified from: Cardillo G, et al. Eur J Cardio-thoracic Surg 2010; 37:581-587.

All patients underwent Chest CT to detect pneumothorax, subcutaneous emphysema. pneumomediastinum, endotracheal tube displacement, and mediastinitis.

Patients with Level I, II or IIIA PITL underwent bronchoscopic application of 1 to 2 ml of fibrin sealant (Tisseel®, Baxter, Deerfield, MA, United States) onto the lesion, covering it with a complete layer. The fibrin sealant was applied through a catheter inserted in the operative channel of the bronchoscope with the endoscopic applicator provided by the manufacturer.

If possible, the procedure was performed with the patient on spontaneous ventilation under local anaesthesia with a flexible bronchoscope. After identification of tracheal laceration, the endoscopic applicator is introduced through the operative channel of flexible bronchoscope, with the distal tip close to the tracheal laceration, then a thin film of fibrin glue was instilled to cover the laceration. If necessary, a second layer of Tiseel was applied.

If the procedure was performed under mechanical ventilation, patients were extubated as soon as clinically indicated. Following endoscopic treatment, antibiotic therapy (as per hospital policy), cough-suppression medication, and total parenteral nutrition were provided to all patients for at least 7 days. Early assessment of bronchoscopic treatment is critical and is mainly based upon clinical signs (improvement of mediastinal/subcutaneous emphysema). On post-operative day 7 a bronchoscopy was routinely performed to confirm PITL healing: If no failure is observed, oral feeding can be initiated and discharge of the patient planned according to clinical course. Follow-up bronchoscopies at outpatient clinics were performed at approximately 28, 90, 180, and 270 days after the operation. Patients with Level IIIB and Level IV PITL underwent immediate surgical repair through thoracotomy, VATS or cervicothomy. Esophageal laceration was commonly repaired by direct running suture. Tracheal injury was repaired with running suture for membranous part, while interrupted single stiches were applied to the cartilaginous part. In case of extensive tissue damage (level IV laceration) tracheal resection of two rings and anastomosis was performed. In this case a muscle flap (sternocleidomastoid muscle) was interposed between trachea and esophagus.

### Data analysis

Statistical analysis was performed using the Stata version 16.1 (STATA corporation, Texas, United States). Continuous variables were expressed in mean and standard deviation (SD) or median and range. Two-tailed Pearson's chi-square test was used for intergroup comparison of categorical variables while the Student *t*-test and Wilcoxon test were used for continuous variables. The boxplot analysis was used for studying the different behaviors of variable of interest in the diverse group. A regression analysis was performed to assess the determinant of PITL grade.

## Results

In our overall series of 62 patients, 11 (17.8%) developed PITL following emergency intubation and 51 (82,2%) after intubation for elective surgery. The mean age of all cohort was 58.2 years (range 12–82).

The majority of PITL cases occurred in females (83.9%, 52/62) compared to males (16.1%, 10/62). According to the body mass index (BMI) the mean value of the entire cohort was 29.9 (range 21–43): 25 patients (40.3%) were overweight (BMI = 25–29.9) and 11 (14%) were obese (BMI > 30). The PITL cases were staged as Level I (*n* = 8), Level II (*n* = 36), Level IIIA (*n* = 11), Level IIIB (*n* = 6), and Level IV (*n* = 1). (Table 2). Bronchoscopic repair with fibrin sealant (Tisseel, Baxter) was performed in all the 55 patients with Level I, II, and IIIA PITL. The 6 patients with Level III B PITL and the patient with Level IV, underwent primary tracheal repair through right posterolateral thoracotomy (5 patients), VATS assisted right anterior minithoracotomy (1), and midline cervicotomy (1).

**Table 2:** Patient characteristics, according to PITL level.

	Level I	Level II	Level IIIA	Level IIIB	Level IV
Cases	8 (12.9%)	36 (58%)	11 (17.8%)	6 (9.7%)	1 (1.6%)
F/M	7/1	32/4	9/2	4/2	0/1
Age (range)	56.7 (53–74)	55 (12–82)	65.2 (50–79)	63.5 (29–77)	62
BMI (kg/m^2^)	26.2	25	28.1	26.3	36.6
BMI <24.9	3	16	4	3	0
BMI >25, < 29,9	3	18	3	1	0
BMI >30	2	2	4	2	1
Intubation: Elective/Emergency	7/1	29/7	11/0	4/2	0/1
Lumen Type: Single/double	4/4	26/10	6/5	3/3	1/0
Single lumen size	7.5 (#3), 8 (#1)	7.5 (#12), 8 (#13), 8.5 (#1)	7.5 (#4), 8 (#2)	7.5 (#1), 8 (#2)	8 (#1)
Double lumen size	35 (#2), 37 (#2), 39 (#1)	35 (#5), 37 (#4), 39 (#1)	35 (#2), 37 (#3)	35 (#1), 37 (#3)	0
Number of rings (range)	4.1 (3–6)	4.6 (2–10)	4.5 (3–8)	4 (3–6)	3
Length of tear [cm] (range)	2.9 (2–3.5)	2.9 (1–6)	2.8 (1.5–4.5)	2.1 (1.5–3.5)	2
Tracheal location (Upper/mid-lower/lower)	1/6/1	7/20/9	1/9/1	0/5/1	0/1/0
Conservative approach (sealant)	8	36	11	0	0
Surgery	0	0	0	6	1
Hospital stay	10	10.6	11.5	17.6	40
Day of antibiotics	7.8	8	8.4	11.5	30
Complications	0	1	2	1	1
Atrial fibrillation		1 (PO day 5)	1 (PO day 7)	1 (PO day 5)	0
Renal failure		0	1 (PO day 4)	0	0
Prolonged intubation		0	0	0	1 (from PO day 0 to PO day 3)

No in hospital (30 day) mortality was reported. Morbidity included atrial fibrillation in 3 patients (1 II 2.7%, 1 IIIA 9%, 1 IIIB 16.6%), renal failure in one patient (1 IIIa 9%), and prolonged intubation in 1 (IIIB). Mean hospital stay was 11.8 (range 7–40) days. None of the 55 patients who received conservative treatment developed mediastinitis after application of fibrin sealant; on day 7 bronchoscopy was routinely performed and showed advanced healing process. Tracheal lesions fully healed within 30 days, without complications. Bronchoscopic evaluations performed 9 months follow-up did not show any tracheal abnormalities. Six of 7 patients who underwent surgical repair had an uneventful recovery with a mean hospital stay of 20.9 days (range 12–40). One patient (Level IV) had a prolonged postoperative intubation. Surgically treated patients were all re-evaluated with Chest-CT before discharged (day fifteen for IIIB, fifteen and thirty in IV).

The patients were then divided into two groups according to PITL grade: group 1 was composed by patients in the PITL stage I and II, and group 2 is formed by patients in the PITL stage IIIA, IIIB and IV.

In Table 3, descriptive statistics for the two groups was presented. The mean age was 55.72 for group 1 and 64.59 for group 2. The mean value of height was not different between the two groups (162.61 cm. for group 1 and 162.06 cm. for group 2). Evaluating the gender in group 1 there were more females than men concerning group 2 (89% for group 1% and 76% for group 2). Finally, the BMI was higher for group 2 (27.46) concerning group 1 (25.87).

**Table 3:** Descriptive statistics across PITL groups.

PITL Group	Age	Height	BMI
**Group 1**			
**Mean**	55,73	162,61	25,87
**SD**	16,69	6,17	3,58
**Min**	12,00	152,00	20,30
**Max**	82,00	180,00	43,00
**Group 2**			
**Mean**	64,59	162,06	27,46
**SD**	12,40	5,86	5,33
**Min**	29,00	149,00	21,00
**Max**	79,00	173,00	40,50
**Total**			
**Mean**	58,20	162,46	26,32
**SD**	16,02	6,04	4,16
**Min**	12,00	149,00	20,30
**Max**	82,00	180,00	43,00

**Figure 3:**
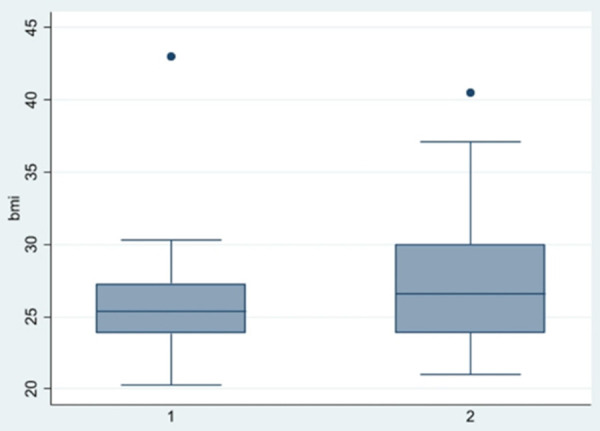
Boxplot BMI across groups.

**Figure 4:**
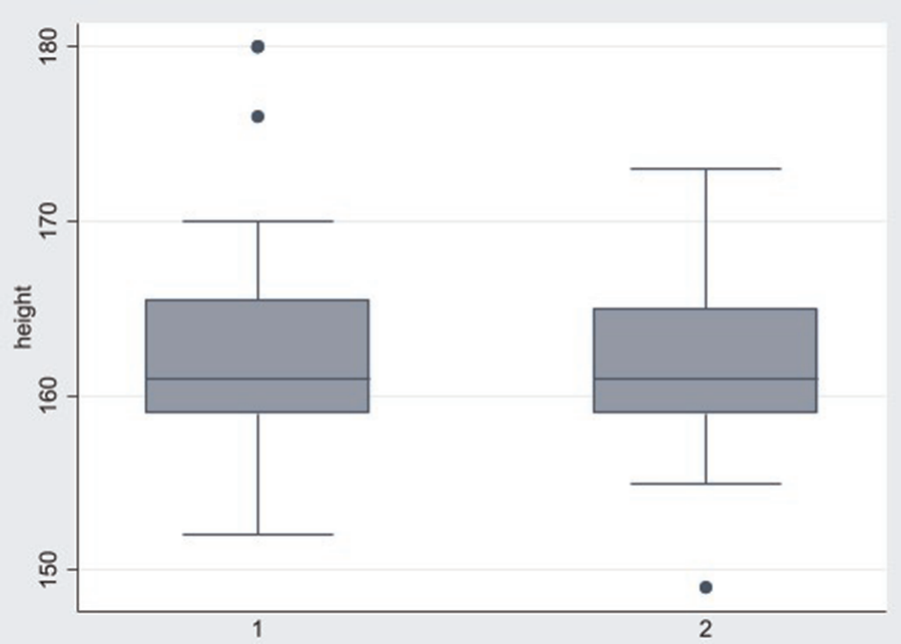
Boxplot height across groups.

**Figure 5:**
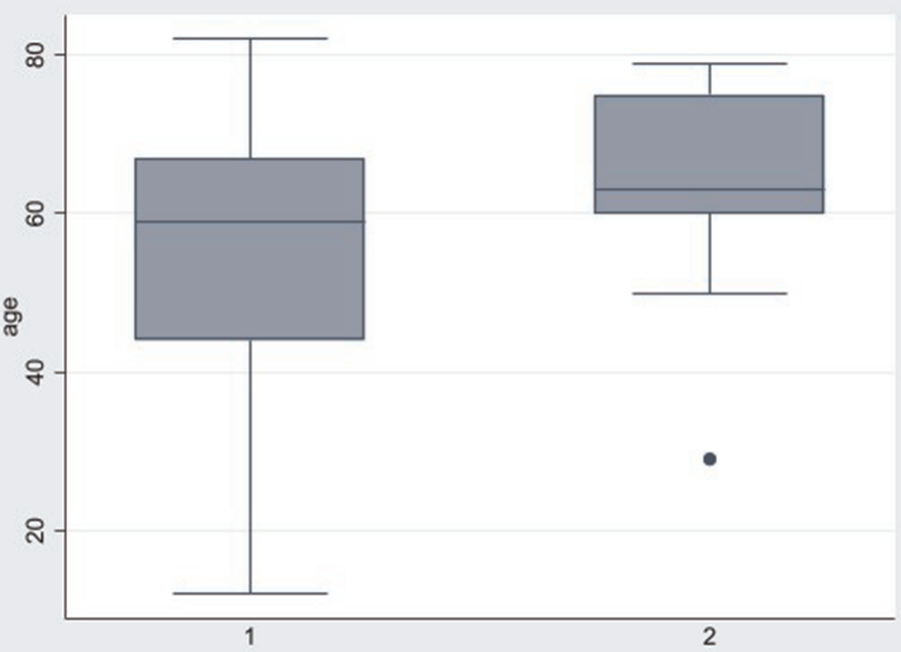
Boxplot age across groups.

In Figure 3, showing the boxplot for BMI, a difference between the two groups was noticed: for group 1, the variance and the median were lower than group 2. Conversely in the boxplot for Height, showing in Figure 4, any difference between the two groups was seen. Finally, analyzing the boxplot for age (Figure 5), we found a significant difference across the two groups (in group 1, the median age of patients is lower than in group 2), and this is consistent with our finding in Table 3.

We implemented Wilcoxon tests (Wilcoxon (1945)) and two-sample *t*-tests to determine whether our variables of interest (BMI, age, and Height) were different across the two diverse PITL group. The null hypothesis for the Wilcoxon test (non-parametric test) was that both distributions were the same, vice-versa the two-sample *t*-test reports the *p*-value for a test for equality of means (Table 4).

**Table 4:** Wilcoxon tests and two-sample *t*-tests on PITL groups (group 1 vs. group2).

	Wilcoxon test		*T*-test	
	Z_statistic	*P* (z)	T_statistics	*p*-value
BMI	−0.76	0.45	−1.13	0.27
Age	−1.92	0.05	−2.26	0.03
Height	−0.09	0.93	0.33	0.75

In most cases, the statistics for the Wilcoxon tests and the *t*-tests were insignificant for both BMI and Height measures, suggesting that these measures were not different across the PITL groups. Notably, there was some evidence that there was a statistically significant difference in age across the diverse PITL group (both for Wilcoxon tests and *t*-test), suggesting that age was an important discriminating factor between the PITL groups.

Finally, we implemented a regression analysis to investigate which is the determinant to be in PITL group 1 or group 2. The empirical results were reported in Table 5. In column (1), we implemented an OLS regression, while in column (2), we implemented a LOGIT regression. The results suggest that an important determinant to be in group 2 was age. Older people were likely to be in group 2 (PITL level IIIA-IIIB-IV).

**Table 5:** Regression analysis.

	OLS	Logit
	(1)	(2)
	PITL	PITL
Age	0.0059*^,^***	0.0369*^,^**
	−1.9677	−1.7006
Height	−0.0144	−0.0759
	(−1.1166)	(−1.0254)
Sex	−0.3511	−1.7158
	(−1.5389)	(−1.4896)
BMI	0.0136	0.0616
	−0.8416	−0.8181
Constant	2.2243	8.9379
	−0.9092	−0.6503
Observations	61	61
R-squared	0.125	

Robust *t*-statistics in parentheses.

* *p* < 0.1.

** *p* < 0.05.

*** *p* < 0.01.

## Discussion

**Figure 6:**
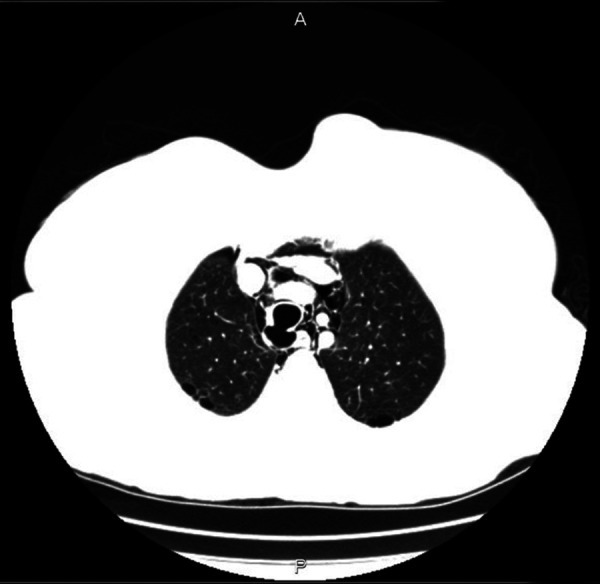
Computed tomography image of level IIIA PITL.

A successful treatment of PITL requires early recognition with proper management according to a risk-stratified protocol which takes into account depth of tracheal involvement as reported in our modified classification (4, 8, 17). Bronchoscopy defines the exact size, site, and extent of the lesion; it can be also used to reposition the tube or to re-intubate the patient if necessary (17–19). The addition, a CT scan can also provide valuable information regarding the PITL and it is mandatory for the proper staging of PITL (Figure 6).

Initial anaesthesiologist approach should be focused on maintenance of cardio-pulmonary stability and assessment of current need for airway management in the non-intubated patient. Spontaneous ventilation should be continued in patients who are not in severe respiratory distress due to concerns for worsening of the injury with positive pressure ventilation or by pressure from an endotracheal tube or its cuff. Coughing should be strongly suppressed, and antibiotic treatment for prevention of mediastinitis should be started.

In a retrospective study of tracheobronchial injuries by Carretta and co-workers, 39 of 50 patients had iatrogenic tracheal injuries; of these, 30/39 patients (76.9%) were treated by open surgical repair while 9 were treated conservatively (23). A surgical approach by thoracotomy, cervicothomy. or VATS carries a high risk of complications, and should be used in selected higher-grade cases (2–9, 13–15, 19, 20, 24–26). Several conservative treatments have been proposed during the years, especially to manage patients unfit for surgery (27, 28). The use of tracheal stents has been described in patients with small tears (<2 cm in length): this approach requires stent explantation six weeks after placement as the risk for occurrence of granulation tissue rises after 3 months. Therefore, the use of tracheal stents seems not to be reliable: no clear indications, high costs and safety not yet been confirmed (2, 4, 6, 18, 21, 22, 29–33).

Most authors have increasingly been choosing and recommending the use of a conservative treatment approach to patients with superficial PITL, using lesion length as a criterion for treatment strategy (1, 4–9, 11–13, 33). However, the identification of PITL cases that would benefit from conservative treatment was not completely defined (1–20) until the publication, in 2010, of our morphological risk-stratified endoscopic classification. It was based on bronchoscopic and CT findings: the depth and not the length of the PITL (11) represents a powerful clinical tool for a risk-stratified therapeutic treatment as recently highlighted in the series by Herrmann (34) and in the review by Singh Grewal (35).

The role of fibrin glue infiltration (Tisseel®, Baxter Healthcare, Deerfield, MA, United States) to promote tissue sealing and regeneration of tracheal lacerations, has not yet been clearly understood (29). Fibrin glue is composed of one component which contains fibrinogen and coagulation factor XIII, and the other containing thrombin dissolved in calcium chloride. Thrombin cleaves fibrinogen, which results in formation of fibrin polymers. In the presence of calcium, thrombin catalyzes conversion of factor XIII to activated factor XIII (factor Xllla). Factor Xllla crosslinks the fibrin polymers into a stable, insoluble fibrin clot (36). We have successfully applied the fibrin sealant to all patients with Level I-IIIA PITL with outstanding results. Probably partial thickness PITL (i.e., Level I, which occurred in 8 cases of our series) may heal spontaneously (14, 2); anyway, since we do not have any control group, we suggest the same approach (3, 19, 20).

Level II PITL is by far the most common tracheal injury (36 out of 62 cases, equal to 58%): careful CT examination is needed before the Level can be confirmed (absence of oesophageal injury or mediastinitis); a prompt and proper endoscopic management, as reported in the present series, is of paramount importance as we can avoid a surgical treatment to the majority of patients with PITL.

Level IIIA, characterized by oesophageal or mediastinal soft-tissue hernia, carries out an additional risk of oesophageal injury or mediastinitis. Conservative treatment can be safe unless there are signs of instability such as respiratory insufficiency, haemoptysis which require emergency surgery at any time. We suggest that these patients should be promptly referred to Units with high competence in thoracic surgery. Level IIIB and Level IV require emergency surgery with no delay.

The use of our modified staging classification reached up to now a 100% success rate for our approach.

An external validation of our morphological classification has been recently added by 2 authors who strictly independently followed our protocol: Herrmann from Germany reported a 97% success rate in a series of 64 patients (34) and Fiorelli from Italy who reported an 83% success rate in 6 pts (36).

The previously published results supplemented by these additional cases and our findings, validates this morphological classification approach to PITL treatment, which showed an overall impressive success rate of 99% (103/104).

Cornerstone of our treatment protocol is early bronchoscopy with treatment initiation within 48 h and close clinical evaluation in the post-operative period. If conservative management fails, surgery should be promptly provided.

Our PITL morphologic classification approach provides clinicians an evidence-based tool which gives the opportunity for treatment selection, offering conservative treatment in patients with stage Level I to IIIA, and surgery in advanced stages (Level IIIB, IV).

A key objective in the management of PITL is mediastinitis prevention. The involvement of oesophagus or the occurrence of a mediastinitis represent a caveat for PITL conservative therapy. In those without evidence of mediastinitis, endoscopic treatment in accordance with our strategies prevents the development of complications. In some instances, especially in stage Level III B and IV advanced life support with extracorporeal membrane oxygenation (ECMO) may be required as a bridge to recovery and/or definitive surgical intervention (34, 35).

The strength of our series is also the balance among the different stages, with most cases being in stage II (36/62; 58%).

The low number of patients surgically treated in our series (7/62; 11.3%); and the zero mortality represent the confirmation of the successful algorithm employed according to our staging system.

Some may question the timing of our follow-up bronchoscopy. In fact, the incidence of long-term tracheal stenosis, which is caused by a retraction phenomenon during recovery after management of PITL, is very low but necessitates a long-term follow-up (i.e., 9 months in our evaluation). We strongly believe that the inclusion of a 9-month bronchoscopy evaluation adds power to the strength of our findings.

A statistical analysis was performed to investigate if gender, BMI, Height and age had an influence on the severity of the PITL. Gender, which is an overall risk factor of developing a PITL (52 out of 62 patients were female) did not show any impact on the severity of PITL score. Height also did not have any influence on the severity of the PITL. BMI and Age showed a difference related to severity of disease in the Boxplot (Figure 4 and Figure 5). Anyway, Wilcoxon tests, the *t*-tests and regression analysis showed advanced age only to be a risk factor: patients with more severe PITL (stage IIIa, IIIB and IV) were older that patients in stage I and II.

## Conclusions

In summary all patients with suspected PITL require immediate (at least < 48 h) bronchoscopy and thoracic surgeon evaluation in order to assess the full extent of the injury, to grade the lesion, and to morphologically individualize the treatment strategy according to our morphological classification of PITL. (11, 34, 36).

Conservative bronchoscopic treatment is effective in patients with partial thickness PITL (level I) or full thickness PITL (level II) with no oesophageal or mediastinal soft-tissue hernia. The presence of oesophageal/mediastinal soft tissue hernia identifies a high-risk category (IIIA) which can be treated conservatively if an adequate respiratory status is achieved and only in highly experienced Centres because of the strong possibility of mediastinitis. Patients with oesophageal injury, mediastinitis (level IIIB) or with extensive loss of substance/fracture of tracheal rings (level IV) requires prompt surgical treatment. The application of fibrin glue on the tracheal laceration helps healing process, even if there are no clear data.

Our 62-patients case-series represent one of the largest experiences in the world literature on the management and outcome of PITL. Additional 42 cases reported from the literature have added external validation to our protocol. We believe that our treatment policy, based upon our proposed morphological classification, represents the gold standard in the treatment of post-intubation tracheal lacerations.

## Data Availability

The raw data supporting the conclusions of this article will be made available by the authors, without undue reservation.
